# Primary Grynfeltt Lumbar Hernia: A Case Report

**DOI:** 10.31729/jnma.7251

**Published:** 2022-02-28

**Authors:** Krity Basnet, Rupa Bhandari, Shiv Raj Shah, Yugal Limbu, Roshan Ghimire

**Affiliations:** 1 Kathmandu Medical College and Teaching Hospital, Sinamangal, Kathmandu, Nepal; 2 Department of Surgery, Kathmandu Medical College and Teaching Hospital, Sinamangal, Kathmandu, Nepal

**Keywords:** *abdominal hernia*, *case report*, *surgical mesh*

## Abstract

A weakening or defect in the posterolateral abdominal wall can lead to development of lumbar hernia. These defects are particularly common in the Petit's inferior triangle or the Grynfeltt-Lesshaft superior triangle. There are very few cases of primary lumbar hernias that have been described in the literature till date. As it is a rare entity, it is often misdiagnosed, leading to delay in management. We present a case of a 66-year-old male with no previous surgery who presented with a mass in the left lumbar region for last ten years. The mass gradually increased in size and caused vague dragging pain. On Computed tomography, the diagnosis of Grynfeltt hernia was made. The patient underwent a laparoscopic mesh repair and had an uneventful postoperative hospital stay. Although a rare entity, there should be a high degree of suspicion of a lumbar hernia when evaluating a case of a lumbar mass. Early diagnosis by computed tomography and management with open or minimally invasive techniques can prevent complications.

## INTRODUCTION

Although abdominal wall hernias have common incidence of 4%-5% globally, lumbar hernias are rare.^12^ It is protrusion of peritoneal contents through a defect in posterolateral abdominal wall,^3^ with 300 reported primary lumbar hernia cases. Hafner stated that surgeons would get only one opportunity in their lifetime to diagnose and operate lumbar hernia.^1^

Lesshaft described superior lumbar hernia in 1870 and Grynfeltt in 1886.^4^ Superior triangle is delineated by 12^th^ rib superiorly, quadratus lumborum medially, internal oblique laterally, floor and roof by external oblique and transversalis fascia.^5^ There is no shielding action of external oblique on transversalis fascia and presence of subcostal neurovascular bundle results in weakness of the triangle.

## CASE REPORT

We report a case of a 66-years old male who presented with a swelling in the left lumbar region for ten years. The swelling was insidious in onset and was gradually increasing in size. The patient also complained of vague dragging pain for the last two years. The patient denied any history of trauma or any other comorbidities.

**Figure 1:**
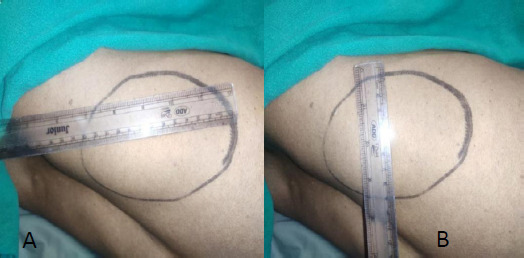
Lumbar hernia lateral view approximately; A) 13cm horizontally, B) 10cm vertically.

On examination, his general condition and vital signs were unremarkable. On local examination, a 10x13cm swelling (Figure 1 A, B) was present in the left lumbar region just below the left 12^th^ rib. It was globular in shape, non-tender, non-mobile, irreducible with a well-defined border and smooth surface. The overlying skin was normal, and a visible cough impulse was present. The swelling was not ballotable and was non-pulsatile.

**Figure 2:**
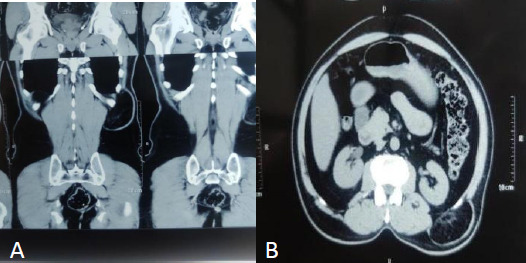
CECT showing left superior lumbar hernia.

Routine laboratory tests were unremarkable. Contrast-Enhanced Computed Tomography (CECT) of Abdomen and Pelvis showed a 4cm defect in the left lumbar region, with herniation of perinephric fat (10x5cm) with no bowel loops suggestive of superior lumbar hernia (Figure 2 A, B).

**Figure 3:**
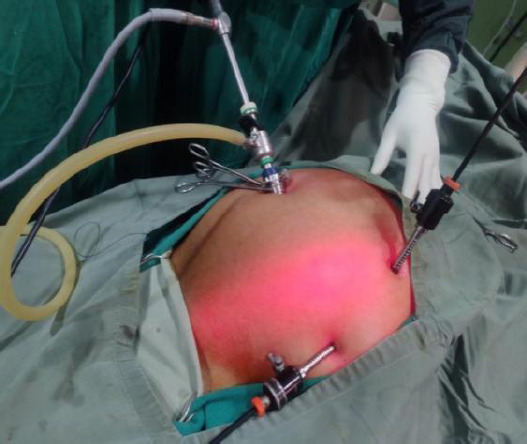
Port placement during laparoscopic approach.

Laparoscopic transperitoneal mesh repair was planned. The patient was kept supine, and pneumoperitoneum was created using Hassan's open technique via a 10mm umbilical port. Two additional ports were created under direct vision in the left hypochondrium and the left iliac region (Figure 3).

**Figure 4:**
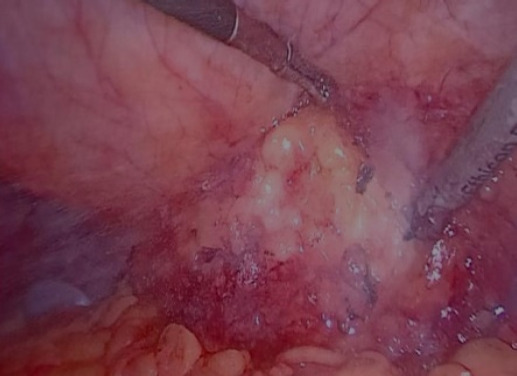
Intraoperative picture showing herniation of the paranephric fat.

The defect was visualized by dissecting omental adhesions and entering the retroperitoneal space by incising the peritoneum over the defect. The location of the defect was also guided by external palpation of the hernia. A left superior lumbar hernia with herniation of perinephric fat was noted intraoperatively (Figure 4).

Prolene mesh of 15x15cm dimension was placed over the defect and fixed with absorbable tackers, followed by the closure of the port sites with polyglactin 910 sutures. The postoperative period was uneventful, and the patient was discharged on the following day. On one week of follow-up, the patient was asymptomatic without any dragging pain in the lumbar region.

## DISCUSSION

Primary lumbar hernia is a rare entity, and the diagnosis in the above case was facilitated by a high degree of clinical suspicion and was confirmed by computed tomography. A lumbar hernia is a protrusion of intraperitoneal or extraperitoneal contents through a defect of the posterolateral abdominal wall.^3^ Increase in intra-abdominal pressure is the most common cause of primarily acquired lumbar hernia, whereas trauma, prior surgical incisions, and abscess formation cause secondarily acquired hernia.

Lumbar hernias can be congenital (20%) or acquired (80%). An acquired hernia can either be primary or secondary. The cause of secondary lumbar hernias can be traumatic or post-surgical following trauma, incision, inflammation comprising about 25% of acquired hernia. Congenital hernias arise from the inferior triangle and can be associated with other anomalies like renal agenesis, lumbo-costovertebral syndrome.^6^ Lumbar hernias are commonly unilateral, occurring on the left side mainly between fifth and seventh decades with a Male:Female ratio of 2:1.^4^

Early detection and management are of paramount importance as up to one out of four cases will become incarcerated, and about one out of ten cases may get strangulated over time.^7^ Patients are usually asymptomatic but can present with dull aching pain over the flank region. At present, there are only few literatures regarding pain management of lumbar hernia though operative being the main line treatment. Somatic pain may present with localized or referred pain to either anterior abdominal wall or through sciatic nerve. The main line management of this pain would be non-opioids agents like non-steroidal anti-inflammatory drugs and acetaminophen. If pain still persists, even after non-opioids treatment weak opioids need to be started.^8^

Mass can be prominent on straining and coughing and reduced on prone position. The patient might also complain of back pain. There can be symptoms of bowel obstruction manifesting with nausea, vomiting, and abdominal distension. If there are renal contents patient presents with urinary complications such as hematuria, oliguria, and colicky pain.^1^ Intestinal obstruction, incarceration, strangulation, and volvulus may complicate lumbar hernias.^6^ Clinical diagnosis maybe difficult, especially in patients who are obese or have non-specific symptoms.

As in our case, the patient presented with left lumbar swelling for the last 10 years with no significant history of trauma or surgeries. On local examination, 10x13cm swelling was present below the 12^th^ rib which was non-tender, non-mobile, irreducible with visible cough impulse. So, lumbar hernia was one of the differential diagnoses in this case. Lumbar hernias are frequently misdiagnosed as lipomas; however, they can also be mistaken for a lumbar hematoma, sarcoma, fibroma, abscess, and renal masses. A Contrast-Enhanced Computed Tomography (CECT) scan is the gold standard for confirming the diagnosis of lumbar hernia.^9^ The diagnosis in our case was also confirmed by CECT of Abdomen and Pelvis when it showed a 4cm defect in left lumbar region with herniation of perinephric fat.

Although lumbar hernia is a rare entity, the surgical procedure for lumbar hernia has constantly been evolving since it was first reported by Owen in 1888.^6^ Only 9% of lumbar hernias present as surgical emergencies while 91% cases are nonemergency cases. Burick AJ, et al. introduced the first trans-abdominal laparoscopic approach.^12^ In Woodward, et al. used balloon dissector for the totally extraperitoneal approach.^13^ A prospective study in between January 1997 and January 2003 of lumbar hernia repairs comparing different approaches, the laparoscopic approach showed statistically significant lower morbidity rates, short hospital stay, reduced analgesics requirement, and early return to everyday activities.^12^ Synthetic mesh for hernia repair may have complications of infection, fistula, and bowel obstruction.

Laparoscopic transperitoneal mesh repair was done in our case. The postoperative hospital stay was uneventful. Follow up was done after a week and the patient was asymptomatic without any dragging pain in the lumbar region. Synthetic mesh for hernia repair may have complications of infection, fistula, and bowel obstruction. Biosynthetic mesh made of the human acellular dermis has been used in some centers, with comparatively good results, especially in contaminated wounds.^1^ Even though various operative techniques are described, there is no consensus on the best approach.^1^ Open hernia repair, which has been used as a conventional approach, is proven safe and effective. With the development of laparoscopic technology, laparoscopic herniorrhaphy is increasingly used to treat a lumbar hernia.^13^ Operative management needs to be decided based on the size of hernia, site, contents, and availability of facilities and expertise of the hospital. For failed laparoscopic approaches and large defects, open surgery may be recommended.^1^ To avoid post operative pain, nerves around superior lumbar triangle such as the ilioinguinal nerve, lateral femoral cutaneous nerve and genitofemoral nerve, needs to be protected, during the operations, especially during fixation.^14^

Primary lumbar hernia is a rare entity and is often misdiagnosed as another lumbar swelling like lipoma, cold TB abscess, and pseudo hernia due to muscular paralysis. A lumbar hernia needs to be considered if the patient presents with pain and swelling over the lumbar region. A Contrast-Enhanced Computed Tomography scan is the gold standard for diagnosing the lumbar hernia. All the lumbar hernia needs to be repaired either by laparoscopic mesh repair or open surgery.

## References

[1] Walgamage TB, Ramesh BS, Alsawafi Y (2015). Case report and review of lumbar hernia.. Int J Surg Case Rep..

[2] Lassandro F, Iasiello F, Pizza NL, Valente T, Stefano ML, Grassi R (2011). Abdominal hernias: Radiological features.. World J GastrointestEndosc..

[3] Stamatiou D, Skandalakis JE, Skandalakis LJ, Mirilas P (2009). Lumbar hernia: surgical anatomy, embryology, and technique of repair.. Am Surg..

[4] Piozzi GN, Cirelli R, Maino MEM, Lenna G (2019). Management Criteria of Grynfeltt's Lumbar Hernia: A Case Report and Review of Literature.. Cureus.

[5] Klingensmith ME, Wise PE (2020). The Washington manual of surgery..

[6] Sundaramurthy S, Suresh HB, Anirudh AV, Prakash Rozario A (2016). Primary lumbar hernia: A rarely encountered hernia.. Int J Surg Case Rep..

[7] Chung I, Wong KY (2019). Bilateral lumbar hernia.. Hong Kong Med J..

[8] Stupalkowska W, Powell-Brett SF, Krijgsman B (2017). Grynfeltt-Lesshaft lumbar hernia: a rare cause of bowel obstruction misdiagnosed as a lipoma.. J Surg Case Rep.

[9] Huang DY, Pan L, Chen MY, Fang J (2018). Laparoscopic repair via the transabdominal preperitoneal procedure for bilateral lumbar hernia: Three cases report and review of literature.. World J Clin Cases..

[10] Kadler B, Shetye A, Patten DK, Al-Nowfal A (2019). A primary inferior lumbar hernia misdiagnosed as a lipoma.. Ann R Coll Surg Engl..

[11] AlAli MN, AlShammari SA, Omar WM, Ayesh M, Alawi K (2019). Bilateral Fat Containing Lumbar Hernia: A Case Report and Literature Review.. Am J Case Rep..

[12] Burick AJ, Parascandola SA (1996). Laparoscopic repair of a traumatic lumbar hernia: a case report.. J Laparoendosc Surg..

[13] Woodward AM, Flint LM, Ferrara JJ (1999). Laparoscopic retroperitoneal repair of recurrent postoperative lumbar hernia.. J Laparoendosc Adv Surg Tech A..

[14] Moreno-Egea A, Torralba-Martinez JA, Morales G, Fernandez T, Girela E, Aguayo-Albasini JL (2005). Open vs laparoscopic repair of secondary lumbar hernias: a prospective nonrandomized study.. Surg Endosc..

